# Adenovirus Infection Is Predicted by Prolonged Duration of Diarrhea among Rotavirus-Vaccinated Children below Five Years of Age in Mwanza, Tanzania

**DOI:** 10.1155/2020/9303216

**Published:** 2020-09-14

**Authors:** Delfina R. Msanga, Tulla S. Masoza, Dina Mahamba, Elizabeth Kwiyolecha, Raphael Rwezaula, Happiness Charles, Regan Kessy, Vitus Silago, Stephan E. Mshana, Mariam M. Mirambo

**Affiliations:** ^1^Department of Pediatrics and Child Health, Weill Bugando School of Medicine, Catholic University of Health and Allied Sciences, P.O. Box 1464, Mwanza, Tanzania; ^2^Department of Pediatrics & Child Health, College of Health Sciences, University of Dodoma, P.O. Box 395, Dodoma, Tanzania; ^3^Department of Microbiology and Immunology, Weill Bugando School of Medicine, Catholic University of Health and Allied Sciences, P.O. Box 1464, Mwanza, Tanzania

## Abstract

Diarrhea is the commonest cause of morbidity and mortality in many resource-limited countries including Tanzania among children below five years of age. A significant number of diarrhea cases associated with severe dehydration are still being reported among children despite five years of rotavirus vaccine implementation in Tanzania necessitating the need to investigate other causes of diarrhea in this population. This study is aimed at determining the prevalence of human adenovirus infection and associated factors among rotavirus-vaccinated children with acute diarrhea in Mwanza, Tanzania. A cross-sectional study was conducted from June to August 2017 involving 137 children less than two years of age admitted with acute diarrhea in the health facilities located in Mwanza, Tanzania. Sociodemographic and other relevant information were collected using standardized rotavirus surveillance tool adopted from WHO. Stool specimens were collected and tested for human adenovirus antigen using immunochromatographic tests. Data were analyzed by using STATA version 13. The median age of enrolled children was 12 (IQR 8-17) months. The prevalence of human adenovirus was found to be 46 (33.6%, 95% CI: 25-41). By multivariable logistic regression analysis, only prolonged duration of diarrhea (OR: 1.619, 95% CI: 1.142-2.295, *p* = 0.007) was found to predict human adenovirus infection among rotavirus-vaccinated children with acute diarrhea. A significant proportion of rotavirus-vaccinated children with prolonged acute diarrhea have adenovirus infection. There is a need to consider other viral pathogens as potential cause of diarrhea especially in this postrotavirus vaccination period.

## 1. Introduction

Diarrheal illness is the second leading cause of mortality in children below five years of age worldwide. Diarrhea causes about 525,000 deaths annually with the majority of these deaths occurring among children below two years [1]. In resource-limited countries, including Tanzania, acute diarrhea has been associated with significant morbidity and mortality as a result of severe dehydration. Globally, the most common etiological cause of acute diarrhea in children below five years of age is rotavirus infection which has been found to cause severe dehydration and prolonged hospital stay [2, 3]. The World Health Organization (WHO) authorized the rotavirus vaccine for infants to be incorporated in all national immunization programme in South-Eastern Asia and in Sub-Saharan Africa, Tanzania being one of them, since 2013 [4]. Despite the five-year implementation of monovalent (G1P8) rotavirus vaccine (Rotarix) in Tanzania, cases of diarrhea associated with severe dehydration among infants are still being reported in a significant number [5]. In Tanzania, G1 was the most predominant G type followed by G8 while P[8] was the most predominant P type, with the frequently common G-P combinations reported being G1P[8] and G1P[6] [6]. This necessitates the need to investigate other viral causes of acute diarrhea in this population or the possibility of other genotypes not covered by the Rotarix vaccine.

Apart from rotavirus infection, adenovirus, norovirus, calicivirus, and astrovirus have been also reported to cause gastroenteritis in childhood [7–9]. Some studies have found that astrovirus was the second most common cause of viral gastroenteritis in infants and young children while others have stressed the importance of adenovirus as the cause of diarrhea sporadically as well as outbreak [10, 11]. There are 88 different human adenovirus (HAdV) types which are grouped into seven HAdV species A to G [12]. Adenoviruses, particularly enteric adenoviruses type 40 (Ad40) and type 41 (Ad41), have been documented to cause acute and severe diarrhea in young children worldwide [13]. In Italy, adenovirus prevalence of 23.2% has been reported among infants with the commonest species being F and C, by 42.4% and 39.4%, respectively [14]. In Africa, the prevalence of adenovirus infection in children with diarrhea has been found to range from 10.4% in Egypt to 37.4% in Kenya [14, 15], while in children without diarrhea the prevalence of 17.6% was reported in Nigeria [13]. In Tanzania, there is limited data regarding the contribution of adenovirus among diarrheal cases in children. This study is aimed at determining the prevalence and associated factors of human adenovirus infection among rotavirus-vaccinated infants with acute diarrhea in the city of Mwanza.

## 2. Material and Methods

### 2.1. Study Design and Duration and Study Area

This was a cross-sectional hospital-based study, conducted from June to August 2017 in three hospitals in the city of Mwanza, Tanzania. These hospitals were Bugando Medical Centre, Sekou Toure Regional Hospital, and Nyamagana District Hospital.

### 2.2. Study Population and Inclusion Criteria

The study included children less than two years of age who were admitted for less than 48 hours due to acute diarrhea. Acute diarrhea was defined as passage of loose, liquid stool more than three times in 24 hours [16]. Prolonged diarrhea was defined as diarrhea lasting for more than seven days.

### 2.3. Sample Size Estimation, Sampling Technique, Inclusion Criteria, and Exclusion Criteria

A sample size was calculated using a Kish Leslie formula using a prevalence of 3.5% from a previous study in Dar es Salaam, Tanzania [17]. All vaccinated children aged 6 weeks to 24 months with acute diarrhea were enrolled serially until the sample size was attained. The study included all rotavirus-vaccinated children admitted for treatment of acute diarrhea with seven days' duration irrespective of the other illnesses and received at least one dose of Rotarix vaccine as evidenced by the RCH card. The study excluded all infants with bloody diarrhea and those who acquired diarrhea during hospitalization.

### 2.4. Data and Sample Collection and Laboratory Procedures

A standardized data collection tool adopted from the WHO surveillance tool was used to collect social demographic and clinical information from consented parents or guardians [18]. Duration of illness, frequency of diarrhea, consistency of stool, and history of rotavirus vaccination were recorded followed by clinical examination to elicit hydration and nutritional status as previously described [19–21]. All admitted children were managed according to the respective standard hospital guidelines. Stool specimen was collected by the parent or guardian using a spatula and placed in a wide-mouth container (HI Media, Mumbai, India). Samples were transported to the multipurpose laboratory CUHAS-Bugando whereby the specimens were processed to detect the presence of adenovirus antigen using immunochromatographic tests (Certest Biotec, San Mateo de Gàllego, Zaragoza, Spain). The assay has sensitivity and specificity of more than 99%. Briefly, fresh stool sample was added into a tube containing 1 mL of diluents and mixed well. Then, 4 to 5 drops (approximately 100-125 *μ*L) of the mixed suspension was put into the sample well of the test device, and results were read after 15 min. All procedures were performed according to the manufacturer's instructions.

### 2.5. Ethical Consideration

The ethical clearance to conduct this study was sought from the joint CUHAS/BMC research ethics and review committee (CREC) and provided with ethical clearance number 630/2018. Permission to conduct the study was sought from the respective hospital administrations. The importance and the protocol of the study were explained to the parents/guardians before a written informed consent was obtained. Confidentiality was maintained throughout the study.

### 2.6. Data Management and Analysis

Data was entered in Microsoft Excel and then transferred to the STATA version 13 for cleaning and analysis. Categorical variables were summarized as proportions while continuous variables were summarized as mean (standard deviation) and median (interquartile range). The Wilcoxon rank sum (Mann–Whitney) test was used to compare the median age of different groups.

Univariable and multivariable logistic regression analyses were used to determine factors associated with adenovirus infection whereby all factors with a *p* value of <0.2 on univariable analysis were subjected into the multivariable analysis. Odds ratio and 95% confidence intervals were determined, and variables with a *p* value of less than 0.05 at 95% CI were considered to have statistically significant difference.

## 3. Results

### 3.1. Sociodemographic and Clinical Characteristics of the Study Participants

A total of 137 children less than 24months were enrolled with a median age of 12 (interquartile range (IQR), 8-17) months. The majority of the children were female 75 (54.7%), and 123 (89.8%) had received three doses of rotavirus vaccine. Among the 137 admitted children, 39 (28.5%) presented with only diarrhea and 98 (71.5%) presented with diarrhea and vomiting. The mean temperature of the enrolled children was 36.5 ± 1.1°C, and 67 (48.9%) had some dehydration at the time of admission. The majority of the children (111, 81%) had no acute malnutrition (Table 1).

### 3.2. Prevalence and Associated Factors of Adenovirus Infection among Rotavirus-Vaccinated Children

Among the 137 enrolled children in this study, 46 (33.6%, 95% CI: 25-41) were found to be infected with adenovirus. By the Wilcoxon rank sum test, there was no significant difference in age (months) between children with adenovirus infection and those without adenovirus infection (4: IQR 3-5 vs. 4: IQR 3-5, *p* = 0.848). Similarly, there was no significant difference in mean body temperature between children infected with adenovirus and those who were not infected with adenovirus (37 ± 0.7°C vs. 37 ± 0.8°C, *p* = 0.919). In addition, there was no significant difference in rates of adenovirus infection between those with only diarrhea and those with diarrhea and vomiting (33.3% vs. 33.6%, *p* = 0.970). By multivariable logistic regression analysis, only prolonged duration of diarrhea (OR: 1.619, 95% CI: 1.142-2.295, *p* = 0.007) was significantly associated with adenovirus infection among rotavirus-vaccinated children with acute diarrhea (Table 2).

## 4. Discussion and Conclusion

Acute diarrhea in children is one of the leading causes of mortality and morbidity in the developing countries. Preventive measures have been taken in Sub-Saharan Africa through introduction of rotavirus vaccine, yet cases of severe diarrhea are being reported in a significant number. Therefore, there is an urgent need to study the epidemiology of other important viruses that are potentially responsible for acute diarrhea for proper management and resource allocation. To the best of our knowledge, this is the first study in Mwanza, Tanzania, to study the contribution of adenovirus infections in acute diarrhea among rotavirus-vaccinated children.

In this study, the prevalence of adenovirus infection in children with acute diarrhea was 33.6%. Similar findings have been observed in Kenya and Nigeria among children less than two years of age [14, 15]. The similarities between these studies could be due to the fact that the population studied was the same based on age group and location; all studies were conducted in urban areas in African countries. In this study, the median age of enrolled children and those infected with adenovirus was 12 (IQR 8-17) months similar to studies in Ghana and coastal Tanzania [2, 17, 22]. It is well known that acute diarrhea affects children less than two years more than older ones. This pattern reflects the combined effects of declining levels of maternally acquired antibodies, lack of active immunity in the infant, direct contact with human or animal feces when the infant starts to crawl, and the introduction of food that may be contaminated with fecal matter [23].

In comparison to previous studies done in coastal Tanzania and Sudan, the reported prevalence of human adenovirus diarrhea in this study is indeed high. Distinct seasonal patterns of diarrhea occur in many geographical areas; the current study was conducted during short raining season which is different from the aforementioned studies conducted in dry warmer seasons [17, 24]. Various reports have mentioned that acute viral diarrhea peaks during short raining season in urban setting and dry cooler months [6, 23, 25]. We speculate that the rainy season in urban settings is often coupled with floods; the floods are partially explained by a poor drainage system in poor resource setting, and as a result, there is enhanced human contact with wastewater, which has been associated with increased transmission of viral and other bacterial infection [26]. Further studies in different seasons of the year are recommended in Mwanza, Tanzania, to understand the epidemiology and transmission of adenovirus especially during postrotavirus vaccination period.

In this study, adenovirus infection was more likely in children with prolonged diarrhea. This is consistent with findings from Australia [17, 27]. Furthermore, we observed that no significant difference was observed regarding dehydration status between those with adenovirus infection and without adenovirus infection. Persistent gastrointestinal symptoms seen in children with adenovirus infection may increase the risk of malnutrition [27].

### 4.1. Limitation

The small sample size may have affected the distribution of factors.

## 5. Conclusion

The prevalence of adenovirus infection is high in the city of Mwanza, Tanzania, and is significantly more in children with prolonged duration of diarrhea. This indicates that after successful implementation of rotavirus vaccine across the country, human adenovirus might be emerging as the commonest etiology of diarrhea among children less than two years in the city of Mwanza, Tanzania. Clinicians should consider adenovirus infections among children with prolonged diarrhea. Routine testing of adenovirus will provide data that will help to understand the epidemiology and clinical course of adenovirus infection and provide education to society on preventive measures.

## Figures and Tables

**Table 1:** Sociodemographic characteristics of the study participants (*n* = 137).

	Number	Percent (%/median)
Age	137	12.66 ± 5.73
		12 (IQR 8-17)
*Sample location*		
BMC	21	15.3
Nyamagana	51	37.2
Sekou Toure	65	47.5
*Residence*		
Rural	6	4.4
Urban	131	95.6
*Sex*		
Female	75	54.7
Male	62	45.3
*Mother/guardian education level*		
Primary	104	75.9
Secondary	33	24.1
*Mother/guardian employment*		
Employed	67	48.9
Not employed	70	51.1
*Duration of diarrhea*	137	4 (IQR 3-6) days
*Consistency*		
Watery	84	61.3
Mucoid	53	38.7
*HIV status*		
Negative	22	16.1
Positive	3	2.2
Unknown	112	81.8
*Temperature*	137	36.5 ± 1.1
*Vomiting*		
No	39	28.5
Yes	98	71.5
*Rehydration before hospital*		
No	70	51.1
Yes	67	48.9
*Previous diarrhea*		
No	89	65.0
Yes	48	35.0
*Neighbor with diarrhea*		
No	115	83.9
Yes	22	16.1
*Weight/height score*		
Moderate malnutrition	11	8.0
Mild malnutrition	15	11.0
No malnutrition	111	81.0
*Rota virus vaccine*		
No	9	6.6
Yes	128	93.4
*Number of rotavirus vaccine received*		
None	9	6.6
One dose	5	3.7
Two doses	123	89.8

**Table 2:** Factors associated with adenovirus infection among children with diarrhea in Mwanza city.

			Univariable analysis		Multivariable analysis	
Variable	Adeno (negative)	Adeno (positive)	Chi square	*p* value	OR (95% CI)	*p* value
	Median % [IQR]	Median % [IQR]				
Age (month)	91 [IQR 8-17]	46 [IQR 8-14]		0.395	0.98 (0.89-1.08)	0.755
*Sex*						
Female	48 [64]	27 [36]				
Male	43 [69.35]	19 [30.65]	0.44	0.509		
*Residence*						
Rural	5 [83.33]	1 [16.67]				
Urban	86 [65.65]	45 [34.35]	0.81	0.370		
*Mother/guardian level of education*						
Primary	68 [65.38]	36 [34.62]				
Secondary	23 [69.70]	10 [30.30]	0.21	0.648		
*Marital status*						
Married	80 [66.12]	41 [33.88]				
Single	11 [68.75]	5 [31.25]	0.04	0.834		
*Duration of diarrhea*	3 [IQR 2-4]	4 [IQR 3-6]		0.002	1.61 (1.14-2.30)	0.007
*Mother/guardian employment*						
Employed	46 [68.66]	21 [31.34]				
Not employed	45 [64.29]	25 [35.71]	0.29	0.588		
*Consistency*						
Watery	57 [67.86]	27 [32]				
Mucoid	34 [64.15]	19 [35.85]	0.20	0.655		
*Temperature*	91 [36.5-37.8]	46 [36.5-37.6]		0.920		
*Rehydration*						
No	49 [70]	21 [30]				
Yes	42 [62.69]	25 [37.31]	0.82	0.365		
*HIV status*						
Negative	13 [59.09]	9 [40.91]				
Positive	2 [66.67]	1 [33.33]				
Unknown	91 [66.42]	36 [32.14]	0.63	0.728		
*Previous diarrhea*						
No	59 [66.29]	30 [33.71]				
Yes	32 [66.67]	16 [33.33]	0.002	0.965		
*Neighbor with diarrhea*						
No	74 [64.35]	41 [35.65]				
Yes	17 [77.27]	5 [22.73]	1.38	0.240		
*Weight/height score*						
Moderate malnutrition	7 [63.64]	4 [36.36]				
Mild malnutrition	10 [66.67]	5 [33.33]				
No malnutrition	74 [66.67]	37 [33.33]	0.04	0.979		
*Dehydration status*						
None	32 [64]	18 [36]				
Severe	19 [73.08]	7 [26.92]				
Some	40 [65.57]	21 [34.43]	0.67	0.716		
*Rotavirus vaccine*						
No	7 [77.78]	2 [22.22]				
Yes	84 [65.63]	44 [34.38]	0.56	0.456		
*First dose*						
0	7 [77.78]	2 [22.22]				
1	2 [40]	3 [60]				
2	82 [66.67]	41 [33.33]	2.09	0.352		

## Data Availability

All data has been included in the manuscript; however, raw data can be obtained upon request to the Director of Research and Publication, Catholic University of Health and Allied Sciences, using email vc@bugando.ac.tz.

## Abbreviations

BMC: Bugando Medical Centre
CI: Confidence interval
CUHAS: Catholic University of Health and Allied Sciences
DNA: Deoxyribonucleic acid
ELISA: Enzyme-linked immunosorbent assay
HAdV: Human adenovirus
ICT: Immunochromatographic test
IQR: Interquartile range
CARS: Coxsackie and adenovirus receptor
OR: Odds ratio
PCR: Polymerase chain reaction
WHO: World Health Organization.
